# Life‐history trade‐offs and environmental variability shape reproductive demography in a mountain ungulate

**DOI:** 10.1111/1365-2656.70137

**Published:** 2025-09-12

**Authors:** Kevin S. White, Taal Levi, Eran Hood, Chris T. Darimont

**Affiliations:** ^1^ Department of Geography University of Victoria Victoria British Columbia Canada; ^2^ Program on the Environment University of Alaska Southeast Juneau Alaska USA; ^3^ Division of Wildlife Conservation (retired) Alaska Department of Fish and Game Juneau Alaska USA; ^4^ Department of Fisheries, Wildlife and Conservation Sciences Oregon State University Corvallis Oregon USA

**Keywords:** climate, life‐history, mountain goat, *Oreamnos americanus*, population ecology, reproduction

## Abstract

1. Alpine ecosystems are changing rapidly with implications for the demography of alpine organisms. Here, we studied a sentinel species of mountain environments—the mountain goat (*Oreamnos americanus*)—to examine hypotheses about intrinsic and extrinsic drivers of reproductive demography using long‐term data collected from individually marked animals across a broad spatiotemporal extent (*n* = 180 females, 3 study areas, 17 years) in coastal Alaska.
2. Our analyses revealed the importance of life‐history trade‐offs and environmental variability on reproductive performance. The cost of reproduction, defined as the impact of reproducing the previous year on the probability of current year parturition, was high, especially for young, largely primiparous females (13%–32% reduction) and old, senescing individuals (27%–43% reduction); parturition of prime‐aged individuals was relatively unaffected (2% reduction).
3. Winter snow accumulation, which alters energetic expenditure and forage availability, exerted strong negative effects on reproduction (20%–35% reduction, depending on age). The relationship between temperature during the preceding summer's growing season and reproduction was likewise negative, although weaker and more variable (10%–15% reduction). Demographic modelling indicated that snow exerted stronger effects on population growth than summer temperature in part due to greater variability in snow versus temperature among years.
4. Our analyses further revealed that reproductive performance did not affect subsequent survival of mothers or offspring, suggesting mountain goats employ a ‘risk‐sensitive’, conservative reproductive strategy that prioritizes survival over reproduction.
5. Taken together, these results fill an important knowledge gap by providing novel insights about the interplay between life‐history trade‐offs and environmental variation, and how they shape the reproductive demography of climate‐sensitive wildlife.

## Graphical abstract

Alpine ecosystems are changing rapidly with implications for the demography of alpine organisms. Through long‐term study, we reveal novel insights about the interplay between life‐history trade‐offs and environmental variation, and how they shape the reproductive demography of climate‐sensitive wildlife.

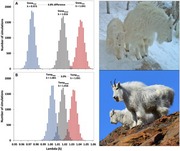

## 1 INTRODUCTION

Reproduction and survival comprise the principal drivers of population dynamics (Gaillard et al., 2000). Whereas survival typically exerts a stronger influence on population growth, reproduction is often more variable (Gaillard et al., 2000), with the potential to overshadow survival‐based contributions in certain contexts (Dulude‐de Broin et al., 2020). These demographic components of fitness can be influenced by a variety of factors, including disease, predation, malnutrition and anthropogenic impacts (Collins & Kays, 2011; Hill et al., 2019). Climate‐linked effects are typically mediated through nutrition‐ and energetic‐based ecological pathways (LaSharr, Dwinnell, et al., 2023; Stephenson et al., 2020), although direct physical processes such as avalanching snow can also have pronounced demographic impacts (White, Hood, et al., 2024). Summer weather conditions are also important and can, for example, influence forage quality and availability (Bø & Hjeljord, 1991; John et al., 2023; Lenart et al., 2002), thermal stress (Mason et al., 2017; Thompson et al., 2020) and insect harassment (Hayes & Berger, 2023). These processes can in turn negatively influence the accumulation of endogenous energetic reserves necessary for survival during winter (Parker et al., 2009), with winter snow playing a major role in affecting nutritional intake through burial of forages and increasing energetic costs of locomotion (Dailey & Hobbs, 1989; White et al., 2009). Thus, through these mechanisms, variation in seasonal weather conditions is expected to exert strong effects on nutritional condition, reproduction and, ultimately, population performance.

In mountain and high‐latitude systems, weather and climate can play a central role in demography (Berger et al., 2018; Desforges et al., 2021; Harris et al., 2024; White et al., 2011). Organisms inhabiting extreme environments are particularly sensitive to changes in environmental conditions (Berger, 2018; Hardie & Hutchings, 2010). Climate is changing more rapidly in mountain regions than in surrounding lowland areas (Pepin et al., 2022), which poses pressing conservation challenges (Lynas et al., 2021; Schmeller et al., 2022). Accordingly, more knowledge is needed to understand potential impacts in these sensitive environments. In alpine ecosystems, a diversity of cold‐adapted species has evolved traits suited for occupying narrow biophysical niches, rendering them particularly sensitive to conditions that deviate outside adaptive norms (Rahbek et al., 2019; Scridel et al., 2018; White, Cadsand, et al., 2024). How variation in weather and climate alters demographic processes carries important implications for the persistence and viability of alpine specialists—many of which are already of conservation concern (Fisher et al., 2022; Jackson et al., 2015; Ray et al., 2013; White et al., 2018).

Mountain ungulates contend with often severe and now rapidly changing environmental conditions, raising questions about their viability under such physical challenges. Study of life‐history theory and trade‐offs may reveal important insights. Life‐history theory provides a framework to examine how individuals balance physiological development, reproduction and survival to optimize fitness (Hutchings, 2021; Stearns, 1998). Life‐history strategies characterize how these components are expressed under constraints imposed by physical limitations and trade‐offs associated with allocation of nutritional resources (Stearns, 1989). The cost of reproduction is a central feature of life‐history theory and relates to how an individual allocates resources to current reproduction relative to ensuring survival (Williams, 1966). Long‐lived, iteroparous species often employ a conservative strategy that favours survival (and the associated opportunity for future reproduction) over investment in current reproduction (Festa‐Bianchet & Côté, 2008; Gaillard et al., 2000; Hamel, Côté, & Festa‐Bianchet, 2010).

Such a conservative strategy is expected to be especially beneficial in seasonal environments characterized by substantial environmental stochasticity. Specifically, allocation of protein and energy reserves is expected to be ‘risk sensitive’ (Bårdsen et al., 2008; LaSharr, Jakopak, et al., 2023; Monteith et al., 2013) and vary with respect to individual nutritional state, relative to seasonal thresholds (Renecker & Samuel, 1991; Stephenson et al., 2020). At the individual‐level, allocation of resources may be adjusted depending on environmental conditions. For example, under harsh or unpredictable environmental conditions, an individual in poor body condition may restrict allocation of fat and protein reserves to reproduction and instead conserve stores to ensure survival, resulting in reproductive pauses, smaller litter size or otherwise reduced investment in offspring (Bårdsen et al., 2014; Bårdsen et al., 2008; Boertje et al., 2019; Monteith et al., 2014; Parker et al., 2009). The capability of individuals to adjust allocation of endogenous nutritional reserves provides an important mechanism for coping with and buffering against environmental extremes, increasing survival and opportunity for future reproduction in tune with risks associated with current reproductive investment (Bårdsen et al., 2008; LaSharr, Jakopak, et al., 2023; Smiley et al., 2022). Thus, the plasticity of life‐history traits allows animals to respond to variability in environmental conditions in ways that allow for optimization of fitness (Hutchings, 2021).

**FIGURE 1:**
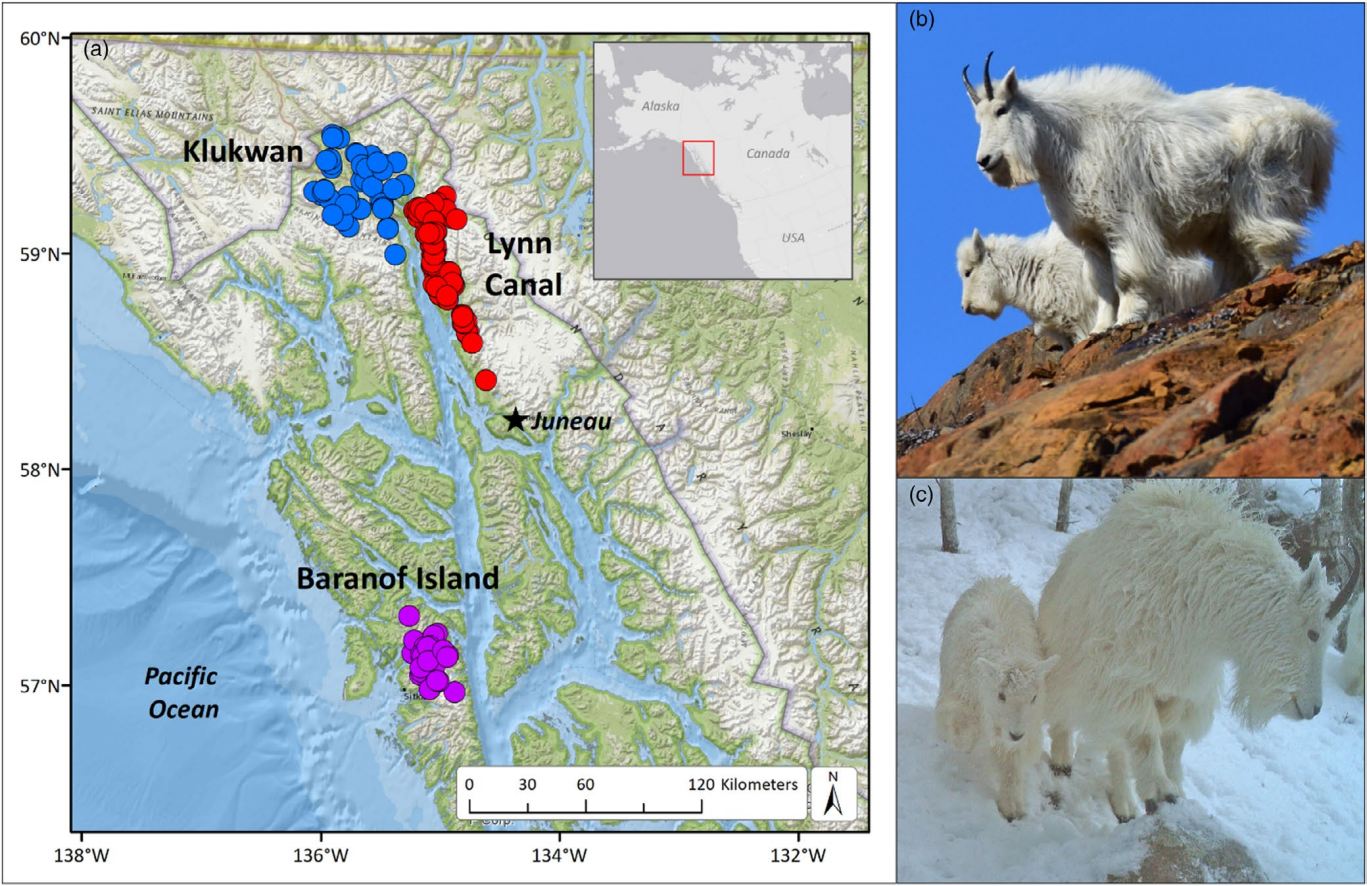
Study system. (a) Map depicting locations where radio‐marked female mountain goats and their offspring were studied during 2005–2021 in three study areas located in coastal Alaska. (b) Female mountain goat (*Oreamnos americanus*) and offspring during early‐spring, experiencing snow‐free conditions following a mild winter. (c) Female mountain goat and offspring in forested winter range during mid‐winter, Takshanuk Mountains, Alaska.

Mountain goats (*Oreamnos americanus*) are sentinels of change in alpine ecosystems, and due to specialized adaptations for life in cold, alpine environments, they are particularly sensitive to changes in weather and climate (Figure 1; White, Cadsand, et al., 2024). The species' distribution spans a wide climatic breadth from the temperate, wet Coast Mountains eastwards to the colder, drier Rocky Mountains, a biogeographic cline that has given rise to behaviourally distinct ‘coastal’ and ‘interior’ ecotypes (Herbert & Turnbull, 1977; Michaud et al., 2024). Much of our knowledge of the species reproductive ecology comes from detailed studies of the interior ecotype (Festa‐Bianchet et al., 2019; Festa‐Bianchet & Côté, 2008). A previous study in coastal Alaska has documented effects of both winter snowfall and summer temperature on survival (White et al., 2011), but how these factors influence reproduction in coastal systems is not well understood. Examining how reproduction is influenced by weather conditions has important practical applications, given the species' sensitivity to climate (Harris et al., 2024; White et al., 2011, 2018) and human disturbance impacts, including harvest (Côté et al., 2013; Hamel et al., 2006; Rice & Gay, 2010). For example, detailed models describing reproductive characteristics can be integrated with existing population models (White et al., 2018; White, Levi, et al., 2021) to predict outcomes of proposed conservation strategies.

Here, we use long‐term (17‐years) longitudinal data collected from individually marked mountain goats (*n* = 180 females) across three coastal Alaska study areas to examine hypotheses about how reproduction is influenced by life‐history trade‐offs and environmental conditions. First, because energetic costs of gestation and rearing young can be substantial (Parker et al., 2009; Stephenson et al., 2020; Testa & Adams, 1998), we predicted that individuals would have a higher probability of giving birth if they had not done so the previous year (Festa‐Bianchet et al., 2019; Hamel, Côté, & Festa‐Bianchet, 2010). We further expected that reproductive performance would differ among life stages that vary in relation to physiological maturity and senescence (Festa‐Bianchet et al., 2019; Lemaître & Gaillard, 2017; Weladji et al., 2010), such that prime‐aged animals would demonstrate lower costs of reproduction than younger and older mothers. We also predicted that survival of mothers and dependent offspring would be similarly affected by previous reproductive investment, but that effects would be weaker, or absent, given the expected prioritization of future survival (and reproductive opportunities) over current reproduction (conservative life‐history strategy; Festa‐Bianchet et al., 2019). In addition, we predicted that reproductive performance would be reduced following periods of environmental stress (i.e. hot summers and snowy winters), paralleling previously documented effects on adult mountain goat survival in this and comparable systems (Harris et al., 2024; White et al., 2011) and among studies of mountain ungulates in other areas (Cosgrove et al., 2021; Rattenbury et al., 2018; Van De Kerk et al., 2020). By extension, we expected variation in life‐history trade‐offs and age‐specific reproduction would likewise be modulated by variation in environmental conditions (Lemaître & Gaillard, 2017; Parker et al., 2009; Stephenson et al., 2020).

## 2 METHODS

### 2.1 Study system

Mountain goats were studied in three areas across a broad geographic range in coastal Alaska (5537 km^2^) from 2005 to 2021 (Figure 1a). This area is within the Coast Mountains biogeographic region (Gallant et al., 1995) and is characterized by a temperate maritime weather and snow climate (McClung & Schaerer, 2006). The region is dominated by coastal temperate rainforest composed primarily of Sitka spruce‐western hemlock (*Picea sitchensis‐Tsuga heterophylla*) forests at lower elevations (below 450–750 m). At higher elevations, subalpine and alpine habitats dominated by krummholtz forest, low‐growing herbaceous meadows and ericaceous heathlands are widespread and persist to elevations of about 1400 m. The geologic terrain is complex and strongly influenced by terrain accretion and uplift processes. Such an arrangement results in a highly fractured landscape dominated by steep and rugged topography that is fragmented by glaciers, icefields, high‐volume river systems and marine waters (Stowell, 2006). Glacier recession has heavily modified the region, leaving steep, sloping topography. Avalanche paths extend from sea level to 2000 m, and impose an important source of landscape disturbance and habitat heterogeneity.

Mountain goats in this region are widespread and occur at low to moderate densities (0.6–1.2/km^2^), typical of northern coastal areas inhabited by the species (Jessen et al., 2022; White et al., 2016). Populations exhibit a high degree of local‐scale population genetic differentiation, with limited movement among geographically discrete mountain complexes (Shafer et al., 2011, 2012; White, Levi, et al., 2021). Mountain goats are habitat specialists and select steep, rugged terrain in close proximity to cliffs and exhibit seasonal variation in altitudinal distribution (Shafer et al., 2012; White et al., 2012; White & Gregovich, 2017). The study populations were partially migratory, with some individuals, depending on the study area, residing in alpine and subalpine habitats throughout the year (Shakeri et al., 2021; White & Gregovich, 2018). However, most individuals conduct short‐distance (5–10 km), seasonal migrations involving annual movements between high‐elevation alpine summer habitats and forested, low‐elevation wintering areas (Shakeri et al., 2021; White et al., 2012; White & Gregovich, 2018). Downslope migrations tend to correspond with the first major snowfall events at high elevation (i.e. mid‐October), while upslope migrations are timed with the onset of snow ablation and the pre‐parturition period (i.e. early‐May) (White et al., 2012). Impacts of human development and activity in the study area are generally minimal. Nonetheless, low‐intensity or localized activities include regulated hunting, ground‐ and air‐based recreational tourism, timber harvest and mining (White & Gregovich, 2017, 2018; White, Levi, et al., 2021). The large mammal predator–prey communities in this area are intact and, in addition to mountain goats, key species include: moose (*Alces alces*), Sitka black‐tailed deer (*Odocoileus hemionus sitkensis*), wolves (*Canis lupus*), coyotes (*Canis latrans*), black bears (*Ursus americanus*), brown bears (*Ursus arctos*) and wolverines (*Gulo gulo*); though local variation occurs relative to species distribution and abundance (MacDonald & Cook, 1996; White et al., 2012).

### 2.2 Mountain goat monitoring

Mountain goats were captured using standard helicopter darting techniques (White, Watts, & Beckmen, 2021). During handling, all animals were fitted with mortality sensing very high frequency (VHF) and/or global positioning system (GPS) radio‐collars (Telonics Inc., Mesa, AZ). The age of animals was determined by counting horn annuli (Brandborg, 1955; Smith, 1988), and, in some cases, cross‐validated by examination of tooth eruption patterns (for young animals) and/or cementum analysis of incisors (for deceased animals; Matson's Laboratory, Milltown, MT). Capture and handling procedures were reviewed and approved by the Alaska Department of Fish and Game Institutional Animal Care and Use Committee (protocols 05‐11, 2016‐25, 0078‐2018‐68, 0039‐2017‐39) and followed American Society of Mammalogists guidelines (Sikes & the Animal Care and Use Committee of the American Society of Mammalogists, 2016).

Following capture, animals were typically monitored at least once per month (often multiple times per month) via aerial telemetry to determine whether collared individuals were alive or dead. Kidding rates and subsequent survival were estimated by monitoring individuals during monthly surveys using fixed‐wing aircraft (usually a Piper PA‐18 Super Cub) equipped for radio‐telemetry tracking or via ground‐based observations. During surveys, radio‐collared adult female mountain goats were observed (typically using 14X image stabilizing binoculars) to determine whether they gave birth to kids and, if so, whether the kid survived until September. Monitoring kid production and survival was only possible during the non‐winter months when animals could be reliably observed in open, alpine habitats. Consequently, we were only able to assess kid survival during the summer period (May–September). Cases in which kid status assessments were equivocal were not used for subsequent estimates.

### 2.3 Winter and summer climate data

We compiled regional climate data from reference weather stations located in the vicinity of Juneau, AK, a geographically central location within 40–100 km of study animals for which weather data are continuously recorded (National Weather Service, Juneau, AK). Following White et al. (2011), temperature measurements were recorded at the Juneau International Airport National Weather Service station and adjusted using the environmental lapse rate (−6.58°C/1000 m; Barry & Van Wie, 1974) to represent the mean elevation (910 m) used by mountain goats in the study region during summer. Summer temperature was expressed as mean daily temperature (C) during July–August during a given year. During the study period, summer temperature averaged 7.89°C (range = 6.00–9.34°C, *n* = 16 years, 2005–2020). Snow measurements were recorded daily at Eaglecrest, Alaska, a mid‐elevation site (366 m) located 9.9 km south of the Juneau Airport, and representative of elevations commonly used by mountain goats in the study region during winter. Snow conditions are expressed as mean daily snow depth (m) during late‐winter (March–April) for a given year. During the study period, snow depth averaged 1.24 m (range = 0.01–2.64 m, *n* = 16 years, 2006–2021).

### 2.4 Data analyses

We evaluated three metrics of mountain goat fitness: parturition success, annual survival of adult females and offspring survival during summer. We used generalized linear mixed effects models (Bolker et al., 2009) to examine the effect of previous reproductive success and environmental conditions on each fitness correlate. Age was also included as a covariate, given that we expected effects to vary depending on individual life stage. In addition, we examined a discrete set of a priori hypothesized interactions focused on assessing whether the effect of previous reproduction depended on life stage and, also, if effects varied depending on environmental conditions. Analyses were conducted separately for each response variable and used reduced subsets of the total data set for which complete longitudinal records were available. In the case of parturition, for example, only cases where previous year and current year parturition were known for a given individual during a given paired year combination comprised a complete record. Incomplete data largely occurred during the year when a female was captured, and knowledge about previous year parturition was unknown or individuals were otherwise not monitored from the beginning of the biological year. Parturition was defined as a binary response variable, as our goal was to estimate the probability of parturition. In practice, such estimates closely match fecundity given twinning is very rare (1.5%, 6/405 cases; see Section 3 below). Survival was also defined as a binary response variable with coding dependent upon whether an adult female survived from June to the following May (termed annual survival) or, in the case of offspring from parturition to September (termed summer survival). Life stages were indexed based on age and encompassed biologically relevant a priori categories spanning primiparous (age: 3 years, age: 4 years, age: 5 years), prime‐aged (age: 6–10 years) and senescent (age: 11–13 years, age: 14–16 years) stages. The primiparous category encompasses the age range in which females are expected to first give birth, whereas the senescent category spans the ranges of ages where reduction in reproductive performance is expected due to old age (sensu Festa‐Bianchet & Côté, 2008). Overall, each age category was coded manually as a dummy variable to simplify the examination of age‐specific interactive effects (i.e. assessment of whether parturition in a given age category was affected differently by a given covariate, as compared to other age categories). A principal goal of our modelling efforts was to understand and parameterize how reproduction and survival varied in relation to age (life‐stage) and, ultimately, whether the effect of other intrinsic and extrinsic covariates on reproduction or survival was age dependent. Thus, we included all age categories in each model considered; though age categories were collapsed, in some instances, if sample sizes precluded adequate parameterization. Overall, we examined hypothesized additive and interactive relationships between parturition, survival of offspring and adults, and age, including as covariates reproductive status, summer temperature and winter snow depth during the biological year preceding parturition (i.e. leading up to and during the gestation period). In the case of adult female survival, however, summer and winter covariate conditions coincided with the current year.

Our modelling approach involved initial examination of random effects considered to be plausible a priori, and included individual identification and site, as well as a term for nesting individual identification within site. However, we included individual identity as a random effect in all models due to repeated measures among individuals across years. We assessed the importance of each random effect by contrasting a global (termed null) model, comprised of only fixed effects terms (GLM), with nested models that included additive effects of each random effect using a generalized linear mixed model (GLMM) framework (logit‐link function and binomial error distribution). We assessed the importance of random effects using an AICc and likelihood ratio tests, based on maximum likelihood estimation (Bolker et al., 2009). Unlike linear mixed models, restricted maximum likelihood (REML) cannot be used for assessing random effects in generalized linear mixed models (Fieberg, 2022). Once the appropriate random effect structure was determined, we used generalized linear mixed models to systematically examine candidate models that a priori represented the hypothesized relationships of interest. We determined the top model(s) by examining the weight of evidence for each model, in comparison with other candidate models, using AICc and by determining whether 95% confidence intervals of *β* coefficient estimates for individual covariates overlapped zero (Burnham & Anderson, 2010). If AICc weights were similar among top models, we considered both models including the more complex and informative models, provided an added parameter(s) was deemed informative per established criteria (sensu Arnold, 2010; Sutherland et al., 2023). We examined model classification and predictive performance by deriving a receiver operating characteristic curve (ROC) and calculating the area under the curve (AUC). We considered AUC values >0.8 to indicate excellent discrimination, 0.7–0.8 to indicate acceptable discrimination, 0.5–0.7 to indicate low discrimination, and <0.5 to indicate poor discrimination (Hosmer & Lemeshow, 2000). We also evaluated the fit between observed versus expected values (QQ plot, K‐S and dispersion tests), and residual plots examining calculated residuals versus predicted values across a range of quantiles. Generalized linear mixed modelling analyses were conducted using *lme4* in R version 4.3.1 (Bates et al., 2014; R Core Team, 2023).

### 2.5 Sensitivity analyses—Climate effects on parturition

We used a population modelling approach to conduct simulations and assess the relative importance of summer versus winter conditions on reproductive performance and ultimately population growth. Specifically, we used a post‐breeding, sex‐ and age‐structured (20 age classes) population model previously described by White et al. (2018) and White, Levi, et al., (2021). Briefly, the model is parameterized using sex‐ and age‐specific survival estimates statistically derived using a spatially and temporally extensive, 44‐year (1977–2021) known‐fates data set collected from mountain goats throughout coastal Alaska (*n* = 14 study sites, 600 individuals, 1910 mountain goat years; White et al., 2011, 2018; White, Levi, et al., (2021), this study). Neonate survival was parameterized following Rice and Gay (2010), as described in White et al. (2018). Age‐specific fecundity was estimated based on direct observations of radio‐marked females using a subset of the data set, as described above.

The original implementation of the model (i.e. White et al., 2018) involved simulating the effects of climatic variability on survival and, ultimately, population growth using average age‐specific fecundity values. In the current analyses, we instead calculate population growth by simulating the effects of variation in summer and winter weather conditions on fecundity, while using average sex‐ and age‐specific survival estimates. Specifically, we examined three scenarios for each weather variable (min, mean, max) and ran 10,000 simulations (30 years time period) for each scenario. For a given scenario, we adjusted the input for the focal climate variable (i.e. winter snow or summer temperature) and held all other inputs at their mean level. To reduce initial transient effects, we initialized each model run (initial population size = 100) based at the stable age distribution, holding all climate variables at their mean level. To account for uncertainty, we sampled from within the error distribution of beta coefficients (i.e. sex‐ and age‐specific survival) accounting for covariance structure among coefficients using the RMark package in R (Laake, 2013). We also modelled interannual variation in annual fecundity as a lognormally distributed random variable. The standard deviation of the distribution was parameterized using the observed variance across the range of interannual fecundity estimates (SD = 0.106, *n* = 16 years, 2006–2021; data described above). This approach enabled us to simulate demographic stochasticity using empirical data collected in our study system.

Overall, our simulation approach enabled us to estimate the mean annual change in population size (*λ*), and its associated distribution, for each scenario (i.e. isolating each climate‐linked reproductive effect on population growth). We then calculated the difference in predicted population growth across the range of variation for winter snow and summer temperature for each scenario. Ultimately, this enabled the determination of the relative strength of each climate driver on population growth due to parturition.

## 3 RESULTS

Overall, we monitored 180 individually marked females across 3 study areas during 2005–2021. We determined annual parturition status on 640 occasions (mean = 3.5 events/individual) among individuals that ranged from 1 to 16 years of age. Individuals were observed with offspring in 405 cases, including 6 instances of twins (1.5% of all parturition events). The proportion of females observed with offspring during the parturition period varied in relation to age. Of parturient females subsequently monitored until autumn (*n* = 311 cases), 84 ± 2% were observed with their offspring. In 24 instances, we commenced monitoring of females between ages 1 and 3 years old and subsequently monitored animals annually (4.6 ± 0.4 years/individual, on average) during the parturition period to determine the age of primiparity. We did not detect any instances of parturition among 1‐ or 2‐year‐old females (Figure S1), with the earliest age of reproduction occurring at 3 years (21%, 5/24 cases). Most females did not give birth until 4 years (54%, 13/24 cases) with the remainder occurring at 5 years (21%, 5/24 cases) and 6 years (4%, 1/24 cases) of age. Overall, the average age of primiparity was 4.1 ± 0.2 (*n* = 24) years (Figure S1).

### 3.1 Modelling age, climate and life‐history trade‐offs

#### 3.1.1 Parturition

To examine the effects of previous year reproduction and weather conditions on the probability of parturition, we examined 13 candidate models describing a priori hypothesized relationships, including relevant interaction terms (Table S1); all models retained individual identity as a random effect. Model selection revealed two top models with near identical AICc weights (Table S1). The top model (AICc *w*_*i*_ = 0.28) included fixed effects for age, previous year reproduction (including a separate interactive effect for prime‐aged animals versus all other age categories) and winter snow. The 2nd best model had near equivalent performance (AICc *w*_*i*_ = 0.27) and was identical except for also including the summer temperature effect. As a consequence, we considered both models but used the more biologically informative, second best model to express relationships between the probability of parturition and the full suite of informative fixed effect parameters.

**FIGURE 2:**
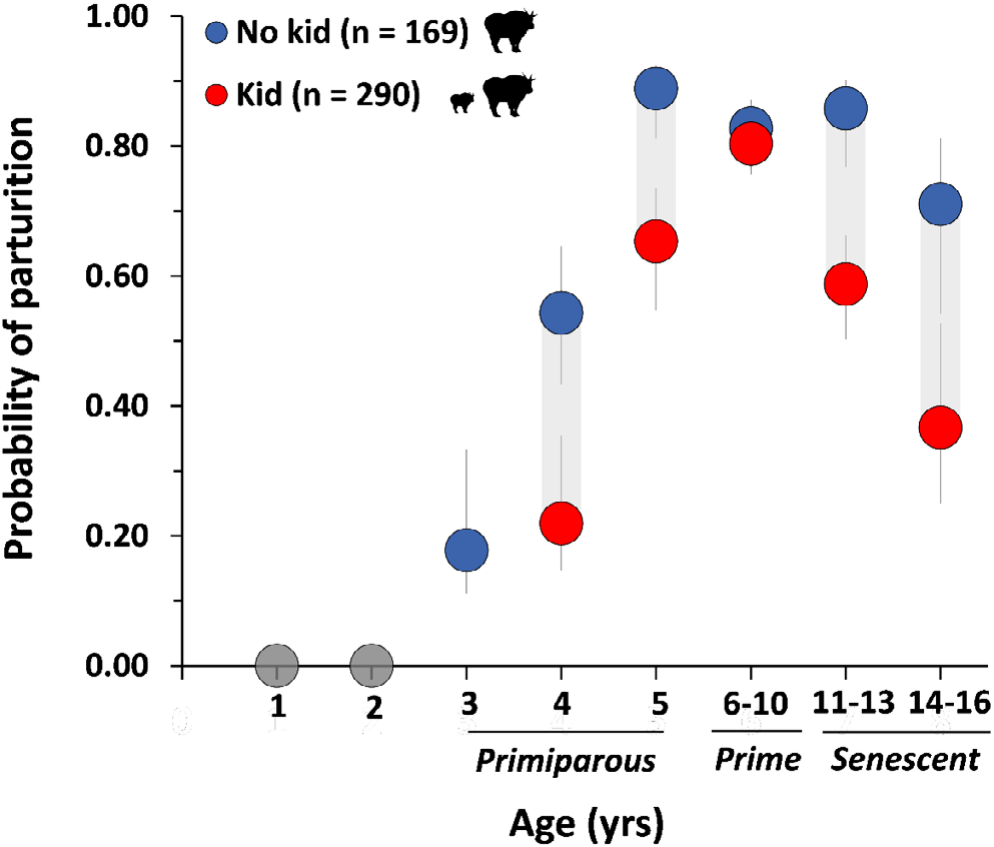
Cost of reproduction in relation to age and previous reproductive performance (no kid/kid) for mountain goats (*n* = 153 females, 459 female years) in coastal Alaska during 2005–2021. Probability of parturition was significantly reduced following successful reproduction for primiparous (age = 3–5 years) and senescent (age = 11–16 years) female mountain goats. A cost of reproduction was not evident for prime‐aged (age = 6–10 years) individuals. Estimates presented for average winter conditions (snow depth, March–April = 1.29 m) and summer conditions (temperature, July–Aug = 7.8°C). Data for 1 and 2‐year olds (*n* = 17 individuals, 21 female years) are plotted on graph (grey circles) for illustrative purposes but were not included in the analysis.

**FIGURE 3:**
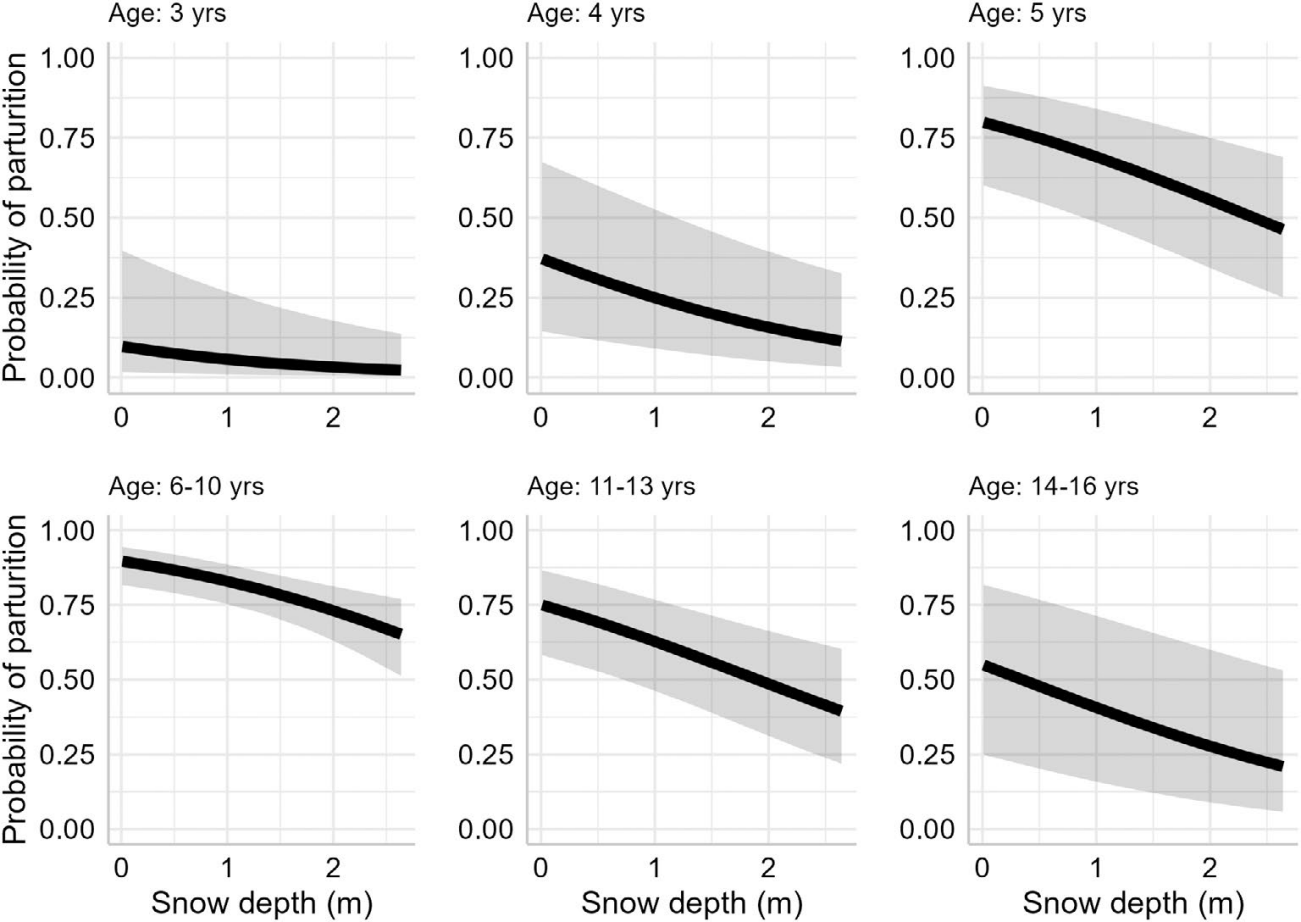
Effect of previous winter snow on age‐specific reproduction for mountain goats in coastal Alaska during 2005–2021. Estimates are presented for individuals that successfully reproduced the previous year. Snow conditions are expressed as mean snow depth (m) during late winter (March–April). Snow measurements were recorded at Eaglecrest, Alaska, a mid‐elevation site (366 m) representative of elevations used by mountain goats in the study region.

**FIGURE 4:**
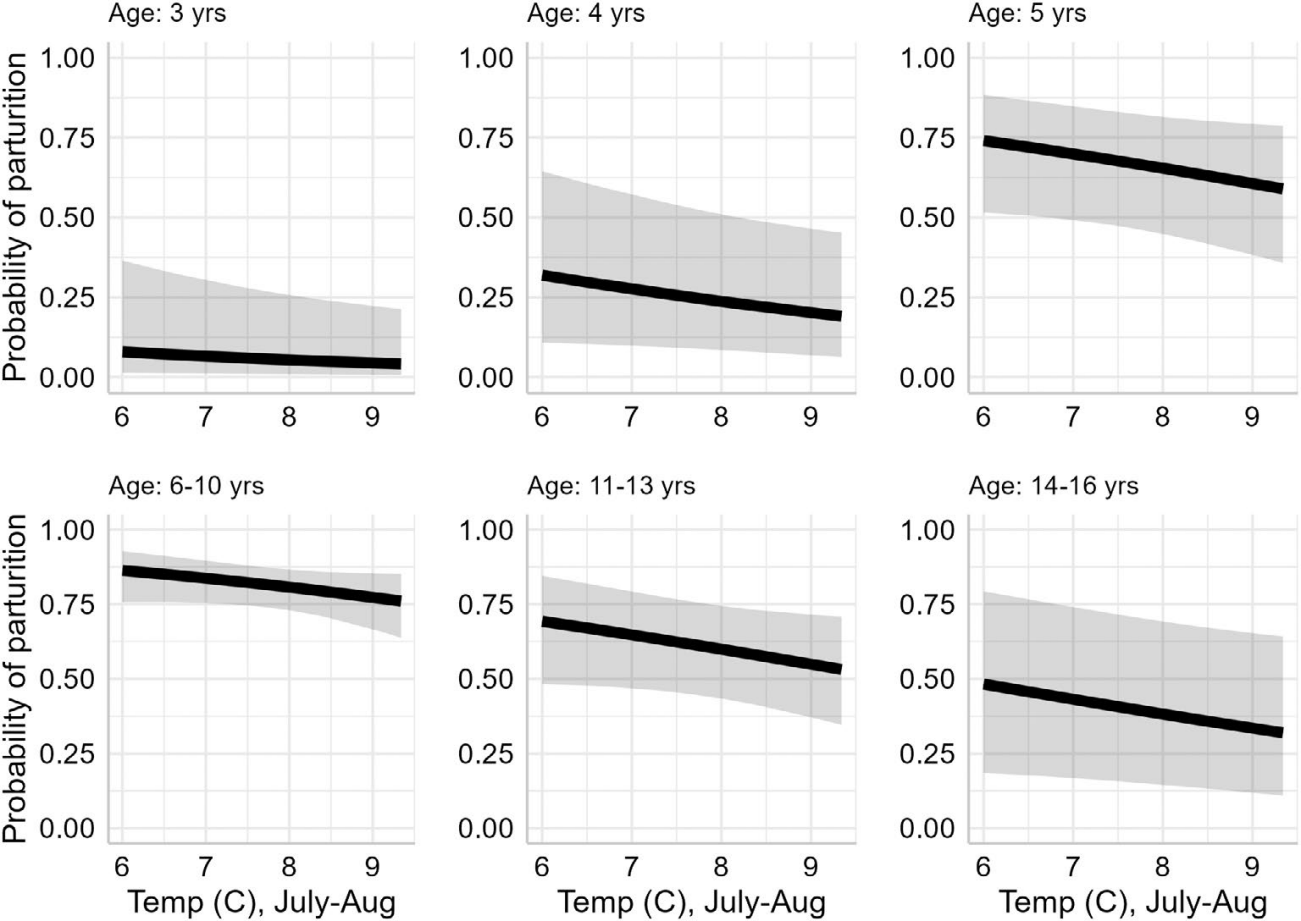
Effect of previous summer temperature on age‐specific reproduction for mountain goats in coastal Alaska during 2005–2021. Estimates are presented for individuals that successfully reproduced the previous year and based on average winter snow depth (1.29 m). Summer temperature is expressed as mean daily temperature (C) during July–August. Following White et al. (2011), temperature measurements were recorded at the Juneau Airport National Weather Service station and adjusted using the environmental lapse rate to represent the mean elevation (910 m) used by mountain goats in the study region during summer.

Specifically, we determined that parturition varied with respect to age, with reproduction being lowest for females within the range of primiparity (termed primiparous) and senescent individuals, and highest for prime‐aged animals (Table 1a, Figure 2). We also documented a strong negative relationship between parturition and the presence of an offspring the previous year (Table 1a, Figure 2). This relationship applied to all age categories consistently, with the exception of prime‐aged individuals. Specifically, parturition of prime‐aged animals was not influenced by giving birth to offspring during the previous year. In addition, we detected a strong negative relationship between parturition and snow depth that consistently affected all age categories (Table 1a, Figure 3). Evidence for an effect of summer temperature on parturition was also negative but was modest in comparison to effects of age, winter snow and previous year reproductive status with the upper confidence interval of the β coefficient estimate marginally overlapping zero (sensu Sutherland et al., 2023; Table 1a, Figure 4). The area under the ROC curve was 0.87 and indicated excellent model discrimination. Further analyses of residuals indicated correspondence between predicted and observed values (Kolmogorov–Smirnov test = 0.55, dispersion test = 0.48).

**TABLE 1:** Effects of age, previous reproductive status and climate on the probability of mountain goat: (a) parturition, (b) adult female annual survival and (c) offspring summer survival in coastal Alaska during 2005–2021.

Parameter	*β*	SE	Confidence intervals		*Z*‐value	*p*‐value
			Lower	Upper		
(*a*) *Probability of parturition*						
Intercept	0.993	1.463	−1.874	3.859	0.679	0.497
Age (4)	1.693	0.844	0.038	3.348	2.005	0.045
Age (5)	3.499	0.927	1.682	5.317	3.773	0.000
Age (6–10)	3.023	0.869	1.320	4.725	3.480	0.001
Age (11–13)	3.264	0.939	1.424	5.105	3.476	0.001
Age (14–16)	2.382	1.038	0.347	4.416	2.295	0.022
Repro_*t*‐1_	−1.386	0.502	−2.370	−0.401	−2.759	0.006
Snow	−0.680	0.187	−1.046	−0.313	−3.635	0.000
Temp	−0.205	0.144	−0.488	0.077	−1.423	0.155
Repro_*t*‐1_ × age (6–10)	1.270	0.569	0.154	2.386	2.230	0.026
(*b*) *Probability of adult female annual survival*						
Intercept	3.315	0.577	2.183	4.446	5.743	0.000
Age (6–10)	−1.164	0.546	−2.234	−0.093	−2.131	0.033
Age (11–16)	−2.127	0.582	−3.267	−0.987	−3.658	0.000
Snow	−0.392	0.185	−0.754	−0.030	−2.123	0.034
(*c*) *Probability of offspring summer survival*						
Intercept	2.206	0.640	0.951	3.460	3.446	0.001
Age (11–16)	−0.703	0.398	−1.484	0.077	−1.766	0.077

*Note*: Age categories are coded as dummy variables with corresponding ages (years) in parentheses. Age categories were combined in some instances to optimize model fit and accommodate sample size limitations. Repro_*t*‐1_ represents parturition status during the previous year. Snow represents average daily snow depth during late‐winter (April–May). Temp represents average daily summer temperature during mid‐summer (July–Aug). Intercepts correspond with: (a) 3 years old females without an offspring the previous year, (b) 3–5 years old females and (c) offspring with mothers aged 3–10 years.

#### 3.1.2 Sensitivity analyses—Climate effects on parturition

**FIGURE 5:**
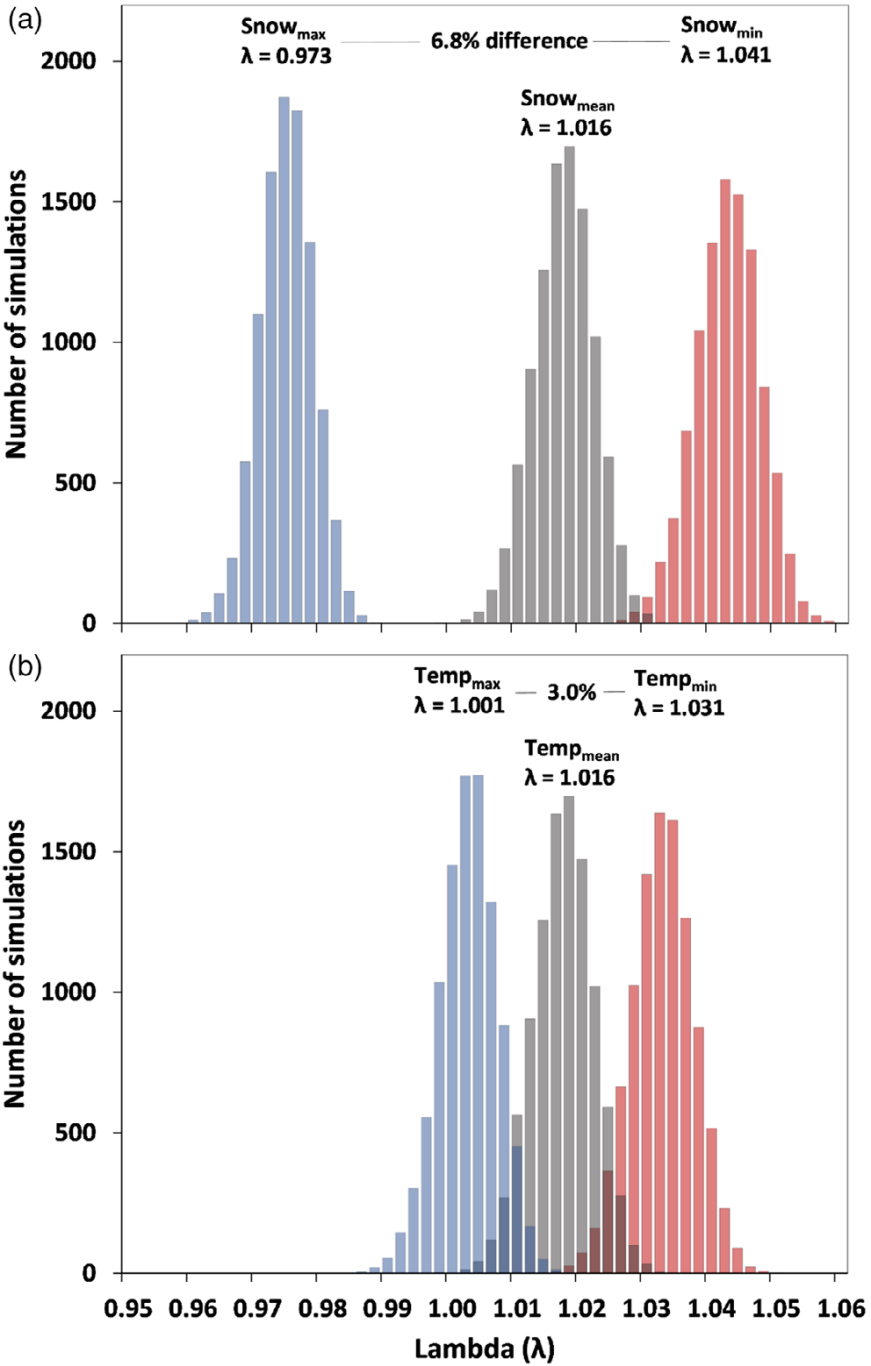
Variation in population growth (*λ*) based on simulated effects of (a) winter snow depth and (b) summer temperature on reproduction across the range of observed climatic conditions. Simulations (*n* = 1000 per scenario), conducted using a sex‐ and age‐specific population model, spanned a 30‐year period and were based on specifying the climate pattern of interest and holding all other input parameters at the mean value.

We used a sex‐ and age‐structured matrix population model to simulate the relative effect of summer versus winter conditions on mountain goat reproduction and ultimately population growth. Our analyses revealed that variation in winter snow exerts a substantially stronger influence on population growth than summer temperature (Figure 5). For example, when all other input parameters were held at the mean value, winter snow had the potential to alter reproductive rates and change annual population growth rate (*λ*) by 6.8%, whereas equivalent assessment of summer temperature revealed a change in *λ* of 3.0%, across the full range of observed conditions. Overall, these differences were attributed to the stronger effect of snow, as compared to summer temperature, on probability of parturition (see above) as well as the greater range of interannual variation in observed mean daily snow depth (min = 0.01 m, mean = 1.21 m, max = 2.64 m) versus mean daily summer temperature (min = 6.00, mean = 7.88°C, max = 9.34) during the 17‐year study period.

#### 3.1.3 Adult female survival

To examine the effects of previous year reproduction and weather conditions on annual survival of adult females, we examined 16 candidate GLMM models describing a priori hypothesized relationships, including relevant interaction terms (Table S2); all models retained individual identity as a random effect to account for repeated measures of individuals across years. We used a reduced subset (*n* = 135 individuals, 343 animal‐years) of our original data set because of the need for information about current year kid status and complete known‐fate survival data for a given year per individual female. We initially considered our original 6‐age category design but also examined structures including 3‐age categories to increase sample size and associated statistical power to parameterize relationships.

The top model (AICc *w*_*i*_ = 17.1) revealed that adult female animal survival varied with respect to age and winter snow depth (Table S2). Specifically, survival was highest among young (age = 3–5 years), intermediate for prime‐aged (age = 6–10 years) and lowest for old (age = 11–16 years) females (Table 1b). Late‐winter snow depth exerted a consistent, negative effect on survival across all age categories. The area under the ROC curve was 0.67, indicating acceptable model discrimination. Further analyses of residuals indicated correspondence between predicted and observed values (Kolmogorov–Smirnov test = 0.46, dispersion test = 0.93). We did not document strong support for effects of previous reproduction or summer temperature on adult female survival. The second best model (AICc *w*_*i*_ = 10.1) was identical to the top model but included previous reproduction. However, the confidence intervals of the *β* coefficient estimate for this variable overlapped zero.

#### 3.1.4 Offspring survival

To examine the effects of previous year reproduction and weather conditions on offspring survival, we examined 13 GLMM candidate models describing a priori hypothesized relationships, including relevant interaction terms (Table S3); all models retained individual identity nested within area as a random effect (i.e. because individuals only occurred within a given study area). We used a reduced subset (*n* = 241 offspring, 116 adult females) of our original data set because of the requirement of having information about the previous year as well as current year offspring status for individual females. We initially considered our original six age category design but also examined structures including three age categories, due to the reduced sample size and associated statistical power to parameterize relationships.

Model selection revealed two competitive top models (Table S3). The top model (Model 3, AICc *w*_*i*_ = 0.23) indicated summer kid survival differed between young/prime‐aged (3–10 years) mothers and old (age 11–16 years) females (Table 1c). Specifically, the probability of kid survival during summer was lower for old mothers (Ŝ = 81.1, CI = 53.4–94.6) as compared to young/prime‐aged females (Ŝ = 90.1, CI = 72.1–97.0). The area under the ROC curve was 0.84, indicating excellent model discrimination. Further analyses of residuals indicated correspondence between predicted and observed values (Kolmogorov–Smirnov test = 0.27, dispersion test = 0.51). The second best model (Model 10, AICc *w*_*i*_ = 0.16) included the same age effect but also an additive effect of snow depth. This model suggested a negative effect of previous winter snow depth on offspring survival during the following summer; but the effect was weak, with confidence intervals overlapping zero. The area under the ROC curve was 0.72, indicating acceptable model discrimination. Further analyses of residuals indicated correspondence between predicted and observed values (Kolmogorov–Smirnov test = 0.11, dispersion test = 0.49).

## 4 DISCUSSION

Knowledge about how vital rates are influenced by variation in environmental conditions and life‐history trade‐offs is important for understanding mechanisms underlying population dynamics. This is especially true for climate‐sensitive mountain species that are vulnerable to the rapid environmental changes currently occurring in alpine ecosystems. Indeed, alpine species spanning an array of taxonomic groups, including birds (Sandercock et al., 2005; Scridel et al., 2018), small mammals (Beever et al., 2011; Morrison & Hik, 2007), carnivores (Fisher et al., 2022; Inman et al., 2012) and ungulates (Jacobson et al., 2004; Van De Kerk et al., 2018; White, Cadsand, et al., 2024) exhibit sensitivity and vulnerability to such change. In this study, we used long‐term longitudinal data spanning a broad spatiotemporal extent and extensive environmental variation to elucidate how mountain ungulate reproduction varied in response to age, life‐history trade‐offs, and changes in summer and winter weather conditions. We found that reproductive performance was sensitive to previous reproductive investment and variation in seasonal weather conditions (especially during winter). Costs of reproduction, however, did not affect maternal survival, highlighting a conservative reproductive strategy. Together with previous work conducted on interior mountain goats (Festa‐Bianchet et al., 2019; Festa‐Bianchet & Côté, 2008; Hamel, Côté, & Festa‐Bianchet, 2010), our study, the first focused on the coastal ecotype of the species, offers a comprehensive understanding of the species' reproductive ecology across a range of conditions and advances our capacity to address conservation challenges faced by the species. Such work is timely given the increasing impacts of climate change on mountain wildlife and alpine ecosystems more broadly (Schmeller et al., 2022; White, Cadsand, et al., 2024).

### 4.1 Life‐history trade‐offs

Reproduction is energetically costly and can invoke trade‐offs dependent on life stage and physical development. Adapted to extreme environmental conditions, mountain goats have slow growth rates and a late age of primiparity, leading to a high cost of reproduction among young animals. In this regard, our findings were similar to other long‐term studies of ungulates that demonstrated that young females had a lower probability of parturition if they had previously given birth, as compared to nulliparous females that had never given birth (Boertje et al., 2019; Festa‐Bianchet et al., 2019; Hamel, Côté, & Festa‐Bianchet, 2010). Such reproductive costs did not occur among prime‐aged individuals, but they were increasingly evident as females entered older age classes. Consistent with the senescence hypothesis (Weladji et al., 2010), evidence of increased costs of reproduction and senescence among older age classes has also been documented among other northern ungulate populations (Boertje et al., 2019; Clutton‐Brock et al., 1983; Ericsson et al., 2001) and across diverse mammalian and avian taxa (Gaillard et al., 2000; Nussey et al., 2013). However, in other instances, factors such as individual heterogeneity, population density and resource availability may overshadow, or modify, age‐specific costs of reproduction (Clutton‐Brock, 1984; Forsythe et al., 2021; Hamel, Côté, & Festa‐Bianchet, 2010; Rauset et al., 2015; Rotella, 2023). Manifested through variation in individual quality, including social rank, body condition, genetics and maternal effects, individual heterogeneity can influence the susceptibility of females to reproductive costs (Forsythe et al., 2021; Hamel, Côté, & Festa‐Bianchet, 2010; Rotella, 2023). Among mountain goats in the Canadian Rockies, for instance, social rank combined with body condition enabled old animals to offset costs of reproduction, as such individuals often had a greater social dominance, larger body mass and apparently greater capability to recover from reproductive events (Hamel, Côté, & Festa‐Bianchet, 2010).

Population density and resource availability can also play a role in limiting reproduction (Clutton‐Brock et al., 1983; Festa‐Bianchet et al., 1998; Rauset et al., 2015; Tavecchia et al., 2005), with reproductive costs being greatest at elevated densities (Boertje et al., 2019; Clutton‐Brock et al., 1983; Hamel, Côté, & Festa‐Bianchet, 2010). When densities are low, however, costs may be evident only among the most physically compromised females, such as young and old individuals, that are unable to adequately recover from reproductive events from 1 year to the next (Clutton‐Brock et al., 1983). Population density in our study areas was relatively low (0.6–1.2 mtn goats/km^2^) and may, in part, explain why we detected reproductive costs in young and old but not prime‐aged females, in comparison to the higher mountain goat population densities studied elsewhere (4.3–5.7 mountain goats/km^2^; Hamel, Côté, & Festa‐Bianchet, 2010) where all females paid a cost of reproduction. Despite nuanced differences in stage‐specific reproductive costs, the general patterns of reproduction including late age of primiparity, very low incidence of twins and frequency of reproductive pauses are consistent among this and previous long‐term studies of mountain goats and provide further empirical evidence of the species' conservative reproductive strategy (Festa‐Bianchet & Côté, 2008).

Given the relatively high energetic cost of reproduction, employing a life‐history strategy that prioritizes survival over reproduction is likely a central adaptation for long‐lived ‘capital’ breeders (Gaillard et al., 2000; Hamel, Gaillard, et al., 2010), especially those that inhabit extreme environments with short growing seasons and unpredictable, often severe, winter conditions (Festa‐Bianchet et al., 1998, 2019). Previous work among northern and mountain ungulates further elucidates these dynamics and suggests that reproduction, by competing with nutritional resources needed for survival, leads to ‘risk sensitive’ reproductive allocation, especially in variable environments (Bårdsen et al., 2008; LaSharr, Jakopak, et al., 2023; Smiley et al., 2022). Our results are consistent with these expectations, revealing that mountain goat reproductive investment and performance is indeed sensitive to variation in environmental conditions and previous parturition success, whereas annual survival of females was not affected by giving birth the previous year. Similar to previous studies among mountain goats (Festa‐Bianchet & Côté, 2008; Hamel, Côté, & Festa‐Bianchet, 2010) and other alpine ungulates (Festa‐Bianchet et al., 2019; Morin et al., 2016; Toigo et al., 2002), these findings indicate that adult females adopt a conservative reproductive strategy that favours their own survival over investment in offspring. Employing a conservative, risk‐averse strategy of reproductive allocation improves the capacity of individuals to buffer against unpredictable environmental conditions (Bårdsen et al., 2008; LaSharr, Jakopak, et al., 2023) and represents a life‐history tactic suited for life in mountain environments often characterized by high climatic variability. Adaptive strategies, however, may have limits. With projected increases in future climate variability, shifts towards increasingly risk‐averse life‐history strategies are expected, but the capacity for species' to adjust life histories to counteract such change is uncertain and may be effective only up to a certain point (Bårdsen et al., 2011; Forcada et al., 2008; Moreno & Møller, 2011).

Maternal costs of reproduction may also impact performance and survival of offspring (Feder et al., 2008; Festa‐Bianchet et al., 2019; Hamel et al., 2009). We expected that offspring born to females that had given birth the previous year would have lower over‐summer survival than those born to females that did not reproduce during the previous year. Such effects can occur when physiological costs of reproduction deplete maternal energetic reserves available for reproductive allocation and translate to reduced physical condition and subsequent survival of offspring (Keech et al., 2000; LaSharr et al., 2025; Testa & Adams, 1998). However, we did not detect evidence of this relationship. Specifically, while we documented a clear cost of reproduction on the future probability of parturition, we determined that such effects did not extend to reducing survival of offspring during their first summer. Similar to findings in interior mountain goat populations (Hamel, Gaillard, et al., 2010), this likely occurs because nutritional constraints are relaxed during the vegetative growing season and effects of previous reproduction on offspring vitality and survival, if present, are not likely to be manifested until the winter season when nutritional deprivation and physiological stress are more pronounced.

### 4.2 Weather and climate effects

Weather and climate represent a principal source of environmental variability and play an important role in shaping the demography of animal populations (Desforges et al., 2021; Felton et al., 2024; Maxwell et al., 2019). As inhabitants of often severe alpine environments, mountain goat demographic processes are particularly sensitive to variation in climate conditions. Previous research has documented negative effects of warm summer temperatures and deep winter snow on mountain goat survival (Harris et al., 2024; White et al., 2011). Our analyses of reproductive performance revealed parallel relationships. Specifically, the probability of parturition declined 10%–15% following the warmest summer conditions, as compared to the coolest. Effects were more pronounced with winter snow depth; we observed a 25%–30% decline in parturition following severe versus mild winters. Translated to population dynamics, our sensitivity analyses indicated that variation in reproduction across the range of observed winter snow depth conditions had greater potential to elicit change in population growth rate (*λ*, range = 0.972–1.041), as compared to variation in observed temperatures during the summer growing season (*λ*, range = 1.001–1.031; Figure 5). Consequently, across the range of conditions observed, winter conditions exerted a stronger effect (in both statistical and absolute terms) on parturition than summer temperature. A similar winter dominant pattern was observed in an earlier, extensive study of winter snow and summer temperature effects on the survival of adult mountain goats in coastal Alaska (White et al., 2011). Across the range of variation observed, for instance, the effect of winter snowfall on adult female survival was nearly twice as strong as summer temperature (White et al., 2011). Taken together, these results suggest similar ecological and physiological mechanisms underlie climate‐linked variation in both reproduction and survival among mountain goats.

Summer and winter weather are expected to principally influence reproductive performance and survival through nutritional pathways that modulate gain and expenditure of endogenous nutritional reserves (Parker et al., 2009). Specifically, during the summer growing season, environmental conditions influence the nutritional characteristics and availability of forage resources (Barboza et al., 2018; John et al., 2023; Lenart et al., 2002; Pettorelli et al., 2007). Ultimately, assimilation of these energy and protein reserves is necessary for survival during winter, when animals experience a negative energy balance due to reduced forage quality, accessibility and increased energetic costs of locomotion (Dailey & Hobbs, 1989; Parker et al., 2009; Stephenson et al., 2020; White et al., 2009). Cold‐adapted northern and mountain ungulates are also particularly sensitive to thermal stress, and during warm summer temperatures, alter behaviour to prioritize staying cool at the expense of optimizing foraging opportunities (Aublet et al., 2009; Mason et al., 2017; Michaud et al., 2024; Thompson et al., 2021). Hot summers can thus present dual challenges, both reducing spatial and temporal availability of high‐quality, early‐phenological stage forages (Fox, 1991; Pettorelli et al., 2007) and constraining optimal habitat use and foraging efficiency (Aublet et al., 2009; Michaud et al., 2024); factors that may be especially important among typically low‐density mountain ungulate populations expected to be more limited by forage quality than forage biomass. When faced with deep, variable or persistent snow packs the following winter, physiological challenges and negative impacts on reproductive performance may be exacerbated for mountain ungulates (Cosgrove et al., 2021; Rattenbury et al., 2018; Stephenson et al., 2020; Van De Kerk et al., 2018), highlighting the important role of seasonal weather conditions and, critically, their temporal sequencing and interrelationships (Mautz, 1978; Parker et al., 2009; Van De Kerk et al., 2020).

That summer and winter weather affect both mountain goat reproduction (this study) and survival (White et al., 2011) in similar ways suggests longer‐term projections regarding climate change impacts on mountain goat population dynamics in coastal Alaska are likely to remain a subject of conservation concern. Specifically, previous survival‐based population modelling simulations indicated the deleterious projected increase in summer temperature was expected to outweigh beneficial declines in winter snowfall and result in long‐term population declines, across a range of general circulation model (GCM) and emissions scenarios (White et al., 2018). This occurs because future climate projections indicate summer temperature and associated effects will continue to increase over time (and extended beyond current observed conditions), whereas change in winter snowfall and associated negative reproductive effects will decrease (Shanley et al., 2015; White et al., 2018). Thus, given the similar directionality among responses of reproduction and survival to summer and winter weather conditions, projected outcomes of climate change on coastal mountain goat population dynamics are expected to remain consistent with, or strengthened, relative to previous simulations.

### 4.3 Ecotypic similarities and conservation implications

Phenotypic traits can vary across geographic gradients within a species distribution, giving rise to distinct ‘ecotypes’ (Lomolino et al., 2017). Among mountain goats, climate and habitat conditions vary along coastal–interior gradients and have led to the suggestion that population performance may likewise differ between coastal and interior regions within the species North American distribution (Herbert & Turnbull, 1977; Rice et al., 2022)—yet empirical evaluation has been limited. Long‐term studies of individually marked animals provide an important opportunity to understand life‐history and reproductive ecology in detail and examine proposed biogeographic relationships. Comparative evidence from long‐term studies of the species in interior (Caw Ridge, Alberta; Festa‐Bianchet et al., 2019) and coastal systems (this study) reveals similar reproductive characteristics, suggesting such adaptations are conserved across their range (Figure S1). Thus, relative to other ungulate species (Gaillard et al., 2000), mountain goats have consistently low reproductive productivity and thus high sensitivity to population perturbations—both natural and anthropogenic. This finding has important practical implications given the diversity of threats faced by mountain goats across their range—impacts that often translate to many alpine species (Sato et al., 2013; Schmeller et al., 2022; Scridel et al., 2018; White, Cadsand, et al., 2024). Climate change, including increased variability and prevalence of extreme events, is a widespread threat for alpine species that, like mountain goats, often have low population growth rates, limited resilience and require long recovery times following decline (Schmeller et al., 2022; White, Cadsand, et al., 2024). Anthropogenic impacts, including industrial and recreational disturbance, landscape change and harvest (Côté et al., 2013; Rice & Gay, 2010; Shackelford et al., 2018; White & Gregovich, 2018), can also be significant and are often additive to climate‐linked stressors (sensu Abrahms et al., 2023). Thus, empirical evidence that key demographic characteristics, such as reproduction, are spatially generalizable across a principal axis of mountain goat distribution has important utility for consideration in broader‐reaching conservation applications.

Species inhabiting extreme environments are particularly sensitive to environmental change and stochastic events, often occurring in small, isolated populations and being disproportionately vulnerable to localized declines or extinctions (Berger, 1990; O'Grady et al., 2004; Turgeon et al., 2024). As such, the implementation of quantitative modelling approaches offers an important tool for understanding population dynamic processes, monitoring population performance and evaluating proposed conservation strategies (Johnson et al., 2010; Mills, 2013; Shenk & Franklin, 2001). The development of models for species, such as mountain goats, that inhabit difficult mountain environments, with long‐term, high‐quality data is logistically challenging but critically needed. Our study provides an important contribution by enabling climate‐varying parameterization of reproductive components for mountain goat population models in the coastal portion of their range. This approach, for example, revealed important insights about the relative effects of summer versus winter weather on mountain goat population growth. More broadly, our analyses and modelling framework advance our capacity for attaining a more comprehensive mechanistic understanding of mountain goat population dynamics in coastal systems. Given the species' sensitivity to climate change and anthropogenic impacts, strengthening analytical tools needed to address conservation challenges represents a promising pathway for ensuring the species' productivity and persistence into the future.

## AUTHOR CONTRIBUTIONS

Kevin S. White: Conceptualization, methodology, investigation, data curation, formal analysis, visualization, funding acquisition, project administration, supervision, writing—original draft, writing—review and editing. Taal Levi: Methodology, investigation, formal analysis, writing—review and editing. Eran Hood: Methodology, investigation, formal analysis, funding acquisition, project administration, supervision, writing—review and editing. Chris T. Darimont: Conceptualization, methodology, visualization, supervision, writing—review and editing.

## CONFLICT OF INTEREST STATEMENT

The authors declare no conflicts of interest.

## STATEMENT ON INCLUSION

Our study brings together authors from the United States and Canada, including scientists based in the country and local areas where the study was conducted. All authors were engaged with the research and study design to ensure that the diverse sets of perspectives they represent was considered from the onset. Whenever relevant, literature published by scientists from the region was cited. Further, outreach with local communities and stakeholders about research activities and findings has been conducted throughout the research process.

## Supporting information


**Table S1.** Model selection results, based on Akaike's Information Criterion with small sample size corrections (AICc), for analyses examining mountain goat parturition in relation to age, winter snow depth, summer temperature and reproductive status during the previous year in coastal Alaska, 2005–2021.
**Table S2.** Model selection results, based on Akaike's Information Criterion with small sample size corrections (AICc), for analyses examining mountain goat adult female annual survival in relation to age, winter snow depth, summer temperature and reproductive status during the previous year in coastal Alaska, 2005–2021.
**Table S3.** Model selection results, based on Akaike's Information Criterion with small sample size corrections (AICc), for analyses examining mountain goat offspring summer survival in relation to maternal age, winter snow depth, summer temperature and reproductive status during the previous year in coastal Alaska, 2005–2021.
**Figure S1.** Age of primiparity and probability of parturition among mountain goats, based on long‐term studies of marked females in coastal southeastern Alaska (2005–2021; this study) and interior Canadian Rocky Mountains (Caw Ridge; 1988–1999; Festa‐Bianchet and Cote 2008).

## Data Availability

Data supporting the results reported in this study are available from the Zenodo repository: https://doi.org/10.5281/zenodo.17027519 (White et al., 2025).
