# Supplementary material for: Life‐history trade‐offs and environmental variability shape reproductive demography in a mountain ungulate
Source: J Anim Ecol. 2025 Sep 12;94(12):2431–47. doi: 10.1111/1365-2656.70137 (PMC12673250; doi:10.1111/1365-2656.70137)

**Supporting Information**

Table S1. Model selection results, based on Akaike’s Information Criterion with small sample size corrections (AICc), for analyses examining mountain goat parturition in relation to age, winter snow depth, summer temperature and reproductive status during the previous year in coastal Alaska, 2005–2021.

| Model | df | AICc | Δ AIC_c_ | weight |
| --- | --- | --- | --- | --- |
| Age + repro_t-1_ + snow + repro_t-1_ 🞨 age(6-10) | 10 | 525.147 | 0.000 | 0.283 |
| Age + repro_t-1_ + snow + temp + repro_t-1_ 🞨 age(6-10) | 11 | 525.236 | 0.088 | 0.271 |
| Age + repro_t-1_ + snow + repro_t-1_ 🞨 age(6-10) + repro_t-1_ 🞨 snow | 11 | 526.522 | 1.375 | 0.142 |
| Age + repro_t-1_ + snow + repro_t-1_ 🞨 age(6-10) + repro_t-1_ 🞨 age(11-16) | 11 | 527.097 | 1.949 | 0.107 |
| Age + repro_t-1_ + snow + temp + repro_t-1_ 🞨age(6-10) + repro_t-1_🞨temp | 12 | 527.340 | 2.193 | 0.094 |
| Age + repro_t-1_ + snow | 9 | 528.221 | 3.074 | 0.061 |
| Age + snow + temp | 9 | 530.063 | 4.916 | 0.024 |
| Age + snow | 8 | 530.789 | 5.642 | 0.017 |
| Age + repro_t-1_ | 8 | 537.598 | 12.450 | 0.001 |
| Age + repro_t-1_ + temp + repro_t-1_🞨age(6-10) | 10 | 537.764 | 12.616 | 0.001 |
| Age | 7 | 539.543 | 14.396 | 0.000 |
| Age + repro_t-1_ + temp | 9 | 539.677 | 14.530 | 0.000 |
| Age + temp | 8 | 541.592 | 16.445 | 0.000 |

Table S2. Model selection results, based on Akaike’s Information Criterion with small sample size corrections (AICc), for analyses examining mountain goat adult female annual survival in relation to age, winter snow depth, summer temperature and reproductive status during the previous year in coastal Alaska, 2005–2021.

| Model | df | AICc | Δ AIC_c_ | weight |
| --- | --- | --- | --- | --- |
| Age(6-10) + Age(11-16) + Snow | 5 | 331.008 | 0.000 | 0.171 |
| Age(6-10) + Age(11-16) + Snow + Repro | 6 | 332.026 | 1.018 | 0.103 |
| Age(6-10) + Age(11-16) + Snow + Repro + Age(6-10) x Repro | 7 | 332.070 | 1.063 | 0.101 |
| Age(6-10) + Age(11-16) + Snow + Age(3-5) x Snow | 6 | 332.325 | 1.317 | 0.089 |
| Age(6-10) + Age(11-16) + Snow + Age(6-10) x Snow | 6 | 332.362 | 1.354 | 0.087 |
| Age(6-10) + Age(11-16) + Snow + Repro + Age(11-16) x Repro | 7 | 332.853 | 1.845 | 0.068 |
| Age(6-10) + Age(11-16) + Snow + Age(11-16) x Snow | 6 | 332.885 | 1.878 | 0.067 |
| Age(6-10) + Age(11-16) + Snow + Repro + Age(3-5) x Snow | 7 | 333.263 | 2.256 | 0.055 |
| Age(6-10) + Age(11-16) | 4 | 333.570 | 2.562 | 0.048 |
| Age(6-10) + Age(11-16) + Snow + Repro + Age(3-5) x Repro | 7 | 333.799 | 2.792 | 0.042 |
| Age(6-10) + Age(11-16) + Snow + Temp + Repro | 7 | 334.037 | 3.029 | 0.038 |
| Age(6-10) + Age(11-16) + Snow + Age(6-10) x Snow + Age(11-16) x Snow | 7 | 334.070 | 3.062 | 0.037 |
| Age(6-10) + Age(11-16) + Snow + Repro + Age(6-10) x Repro + Age(11-16) x Repro | 8 | 334.157 | 3.149 | 0.035 |
| Age(6-10) + Age(11-16) + Repro | 5 | 334.697 | 3.689 | 0.027 |
| Age(6-10) + Age(11-16) + Temp | 5 | 335.331 | 4.323 | 0.020 |
| Age(6-10) + Age(11-16) + Temp + Repro | 6 | 336.392 | 5.384 | 0.012 |

Table S3. Model selection results, based on Akaike’s Information Criterion with small sample size corrections (AICc), for analyses examining mountain goat offspring summer survival in relation to maternal age, winter snow depth, summer temperature and reproductive status during the previous year in coastal Alaska, 2005–2021.

| Model | df | AICc | Δ AIC_c_ | weight |
| --- | --- | --- | --- | --- |
| Age(11-16) | 4 | 225.297 | 0.000 | 0.232 |
| Age(11-16) + Snow | 5 | 226.035 | 0.738 | 0.160 |
| Age(11-16) + Repro_t-1_ | 5 | 227.012 | 1.716 | 0.098 |
| Age(6-10) + Age(11-16) | 5 | 227.215 | 1.918 | 0.089 |
| Age(11-16) + Temp | 5 | 227.217 | 1.920 | 0.089 |
| Age(11-16) + Snow + Repro_t-1_ | 6 | 227.750 | 2.453 | 0.068 |
| Repro_t-1_ | 4 | 227.778 | 2.482 | 0.067 |
| Age(11-16) + Snow + Temp + Repro_t-1_ | 7 | 228.603 | 3.306 | 0.044 |
| Age(4) + Age(5) + Age(6-10) + Age (11-13) + Age(14-16) | 8 | 228.747 | 3.450 | 0.041 |
| Age(11-16) + Temp + Repro_t-1_ | 6 | 228.971 | 3.674 | 0.037 |
| Age(11-16) + Repro_t-1_ + Age(11-16) x Repro_t-1_ | 6 | 229.036 | 3.740 | 0.036 |
| Age(11-16) + Snow + Repro_t-1_ + Snow x Repro_t-1_ | 7 | 229.776 | 4.479 | 0.025 |
| Age(11-16) + Temp + Repro_t-1_ + Temp x Repro_t-1_ | 7 | 230.768 | 5.471 | 0.015 |

Figure S1. Age of primiparity and probability of parturition among mountain goats, based on long-term studies of marked females in coastal southeastern Alaska (2005-2021; this study) and interior Canadian Rocky Mountains (Caw Ridge; 1988-1999; Festa-Bianchet and Cote 2008).


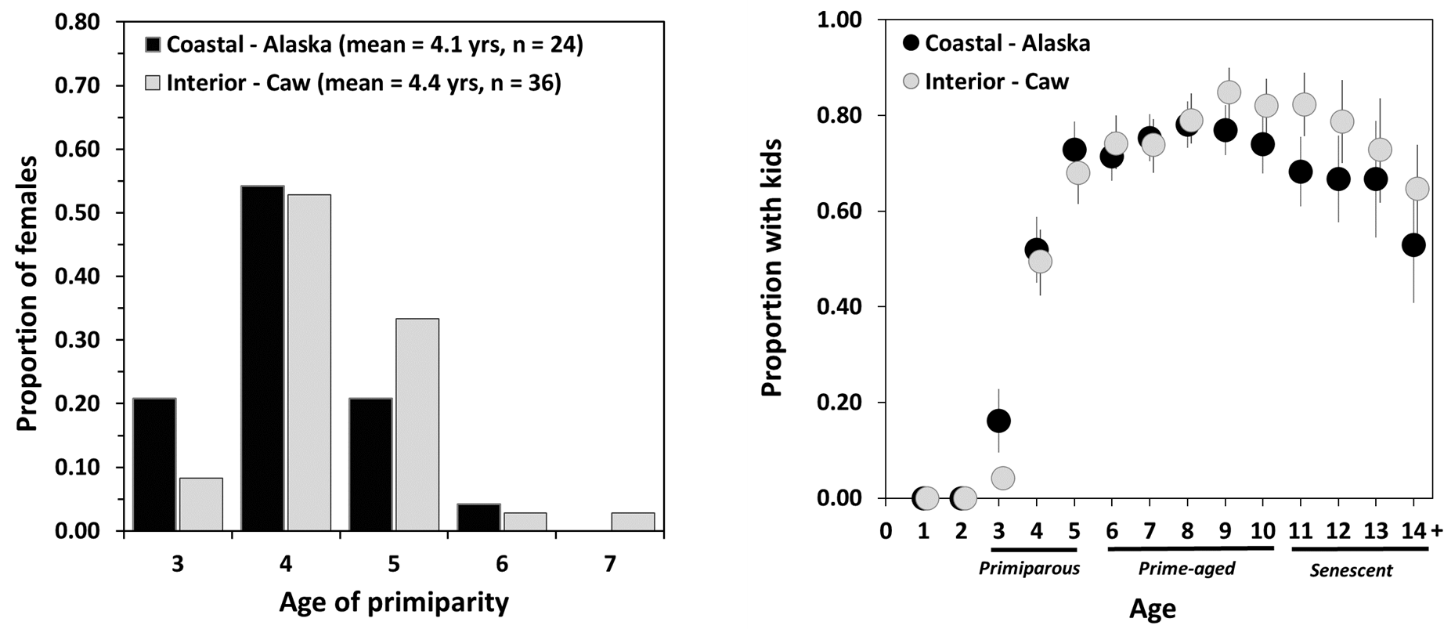

Supplement: Supplementary file 1 — Table S1. Model selection results, based on Akaike's Information Criterion with small sample size corrections (AICc), for analyses examining mountain goat parturition in relation to age, winter snow depth, summer temperature and reproductive status during the previous year in coastal Alaska, 2005–2021. Table S2. Model selection results, based on Akaike's Information Criterion with small sample size corrections (AICc), for analyses examining mountain goat adult female annual survival in relation to age, winter snow depth, summer temperature and reproductive status during the previous year in coastal Alaska, 2005–2021. Table S3. Model selection results, based on Akaike's Information Criterion with small sample size corrections (AICc), for analyses examining mountain goat offspring summer survival in relation to maternal age, winter snow depth, summer temperature and reproductive status during the previous year in coastal Alaska, 2005–2021. Figure S1. Age of primiparity and probability of parturition among mountain goats, based on long‐term studies of marked females in coastal southeastern Alaska (2005–2021; this study) and interior Canadian Rocky Mountains (Caw Ridge; 1988–1999; Festa‐Bianchet and Cote 2008). [file JANE-94-2431-s001.docx]
